# Phenotypic Modulation of Cultured Primary Human Aortic Vascular Smooth Muscle Cells by Uremic Serum

**DOI:** 10.3389/fphys.2018.00089

**Published:** 2018-02-12

**Authors:** Violeta Cazaña-Pérez, Pilar Cidad, Javier Donate-Correa, Ernesto Martín-Núñez, José R. López-López, M. Teresa Pérez-García, Teresa Giraldez, Juan F. Navarro-González, Diego Alvarez de la Rosa

**Affiliations:** ^1^Departamento de Ciencias Médicas Básicas (Fisiología), Instituto de Tecnologías Biomédicas and Centro de Investigaciones Biomédicas de Canarias, Universidad de La Laguna, Tenerife, Spain; ^2^Unidad de Investigación, Hospital Universitario Nuestra Señora de Candelaria, Tenerife, Spain; ^3^Departamento de Bioquímica y Biología Molecular y Fisiología e Instituto de Biología y Genética Molecular (IBGM), Universidad de Valladolid y Consejo Superior de Investigaciones Científicas (CSIC), Valladolid, Spain

**Keywords:** vascular calcification, uremia, chronic kidney disease, apoptosis, osteogenic differentiation, uremic serum, inorganic phosphate, human aorta

## Abstract

Patients with chronic kidney disease (CKD) have a markedly increased incidence of cardiovascular disease (CVD). The high concentration of circulating uremic toxins and alterations in mineral metabolism and hormone levels produce vascular wall remodeling and significant vascular damage. Medial calcification is an early vascular event in CKD patients and is associated to apoptosis or necrosis and trans-differentiation of vascular smooth muscle cells (VSMC) to an osteogenic phenotype. VSMC obtained from bovine or rat aorta and cultured in the presence of increased inorganic phosphate (Pi) have been extensively used to study these processes. In this study we used human aortic VSMC primary cultures to compare the effects of increased Pi to treatment with serum obtained from uremic patients. Uremic serum induced calcification, trans-differentiation and phenotypic remodeling even with normal Pi levels. In spite of similar calcification kinetics, there were fundamental differences in osteochondrogenic marker expression and alkaline phosphatase induction between Pi and uremic serum-treated cells. Moreover, high Pi induced a dramatic decrease in cell viability, while uremic serum preserved it. In summary, our data suggests that primary cultures of human VSMC treated with serum from uremic patients provides a more informative model for the study of vascular calcification secondary to CKD.

## Introduction

Chronic kidney disease (CKD) is a highly prevalent condition characterized by gradual reduction in renal function over time. CKD can arise from a variety of situations that affect the structure and function of the kidney, including advanced age, hypertension, diabetes, or cardiovascular disease (Levey and Coresh, 2012). Decreased renal function, measured as declined glomerular filtration rate (GFR), is tightly linked to increased cardiovascular risk (Tonelli and Pfeffer, 2007). During end-stage renal disease plasma accumulation of metabolites normally cleared by the kidney create a condition known as uremia (Meyer and Hostetter, 2007). Disorders of mineral metabolism, including alterations in the normal homeostasis of calcium, phosphorus, vitamin D, and parathyroid hormone, are an universal complication of end-stage renal disease (Meyer and Hostetter, 2007; Levey and Coresh, 2012).

Increased cardiovascular risk associated to CKD and uremia includes both cardiac and vascular components (Tonelli and Pfeffer, 2007). Hemodynamic changes and the altered metabolic state facilitate vascular wall remodeling resulting in structural and functional abnormalities. Atherosclerosis in renal patients is distinct from that in subjects without renal dysfunction, and it has been described as severe and accelerated, with increased calcification of atheroma plaques (Schwarz et al., 2000; Lu et al., 2014). *In vitro* experiments with arteries from uremic patients have demonstrated special characteristics of arteriosclerosis, with fibroelastic media and intima thickening, increased number of vascular smooth muscle cells (VSMC), increased extracellular matrix volume and increased calcification (Amann et al., 2003). Patients with CKD show intimal and medial calcification, with simultaneous occurrence of both processes in the same patient (Ibels et al., 1979; Schwarz et al., 2000).

Intimal calcification is associated with areas of atherosclerotic plaque, where takes place the combination of necrosis, inflammation and deposition of cholesterol, phospholipids, and lipoproteins (Boström et al., 1993; Demer, 2002). However, medial calcification is an early vascular event in CKD (Moe and Chen, 2004; Benz et al., 2017), which is directly triggered by uremia and alterations in mineral metabolism (Chen et al., 2006b). It has been demonstrated that this condition is not a passive deposition of mineral salts in the vascular wall, but rather a complex and highly regulated phenomenon by which VSMCs suffer an osteochondrogenic trans-differentiation process, eliciting expression of ossification proteins (Chen et al., 2006a) such as alkaline phosphatase, and increased expression of osteochondrogenic factors such as RUNX2 or MSX2, and chondrocyte-specific factors such as SOX9 (Giachelli, 2004).

Increased circulating phosphate concentration is a common finding in CKD patients (Lu et al., 2014). In cultured VSMC addition of phosphate to achieve levels similar to those found in CKD patients (>2 mM) induce mineralization foci with characteristics similar to those found *in vivo* (Jono et al., 2000; Giachelli, 2004; Lu et al., 2014). It has been proposed that the Na^+^-dependent phosphate transporter Pit-1 allows for the accumulation of Pi in the cell, which in turns triggers trans-differentiation (Crouthamel et al., 2013). However, Pi transport in VSMC is saturated under normal conditions and Pit-1 expression does not appear to be increased under hyperphosphatemia, indicating that other factors may provide additional signals to induce osteochondrogenic differentiation and calcification (Villa-Bellosta et al., 2007; Villa-Bellosta and Sorribas, 2009).

Many aspects regarding the complex process of vascular calcification and phenotypic remodeling secondary to CKD and uremia remain unanswered. The process has been studied using different *in vitro* models, including intact vessels (Shroff et al., 2008), aortic rings (Lomashvili et al., 2004; Sonou et al., 2015), or dispersed VSMC, mainly obtained from bovine or rat thoracic aorta (Chen et al., 2006a; Villa-Bellosta et al., 2007; Hortells et al., 2015). In spite of the limitations associated with cultured VSMC, such as the lack of extracellular elastin fibers (Lin et al., 2011) and the diversity of phenotypes that can co-exist in culture (Patel et al., 2016), this model has been extensively used to study vascular calcification. Particularly, the fact that increased Pi in the culture medium induces mineralization and increases osteoblastic markers makes the VSMC model very popular in calcification research. However, it has been demonstrated that small changes in culture conditions can produce significantly different results (Hortells et al., 2015). In addition, it has now become clear that even though Pi is a significant player in the process, other metabolites present in uremic serum are involved in the structural and functional alteration of the vascular wall in CKD (Smith, 2016; Yamada and Giachelli, 2017). Finally, species-specific differences may difficult the reproducibility of observations in the field (Scialla et al., 2013).

In this study we used human aortic VSMC primary cultures treated with serum from uremic patients or healthy individuals, and compared them with cultures with increased Pi concentrations to study calcification and phenotypic remodeling. Our results showed that uremic serum is able to induce calcification, trans-differentiation and phenotypic remodeling even with normal Pi levels. Even though the kinetic of calcification induced by serum was similar to that observed with increased Pi, analysis of molecular markers revealed fundamental differences in osteochondrogenic marker expression and alkaline phosphatase induction. Moreover, high Pi induced a dramatic decrease in cell viability, while human uremic serum preserved it, with a modest increase in apoptosis but a drastic change in phenotype.

## Materials and methods

### Ethical approval, patient selection, and serum biochemistry

Patient and healthy individuals were recruited at the Nephrology Service, Hospital Universitario Nuestra Señora de Candelaria (Tenerife, Spain) after approval by the Hospital Ethics Committee. All subjects gave informed consent to participate. The studies conformed to the standards set by the latest revision of the Declaration of Helsinki.

Control serum samples were obtained from 16 healthy donors and uremic serum samples were obtained from 16 patients with stage 5D CKD (estimated GFR ≤ 15 ml/min/1,73 m^2^). Characteristics and biochemical parameters of healthy (control) and uremic serum samples are summarized in Table 1. All participants were older than 18 years old. Exclusion criteria included pregnancy, current smoking habit, alcohol dependence or drug abuse, history of immunologic, or tumoral disease, an acute inflammatory or infectious episode in the previous month, hepatitis B, C, or HIV positivity, prior transplantation, and immunotherapy, immunosuppressive, anti-inflammatory, or steroid treatment. Blood samples were obtained after an 8 h fasting period by venipuncture. After centrifugation aliquots of individual serum samples were saved for biochemical determinations and the rest were pooled, aliquoted and stored at −80°C until use. Each sample was thawed only once.

**Table 1:** Characteristics and biochemical parameters of healthy (control) and uremic serum.

	**Control serum**	**Uremic serum**	***p*-value**
Number of patients	16	16	–
Age (years)	39–58	25–45	
Gender (male;female)	6;10	8;8	–
Albumin (mg/dl)	4.1 ± 0.07	3.81 ± 0.1	0.005
Urea (mg/dl)	33.5 ± 1.8	88.56 ± 4.2	<0.0001
Creatinine (mg/dl)	1.01 ± 0.0	5.38 ± 0.2	<0.0001
Inorganic phosphate (mg/dl)	3.57 ± 0.1	5.84 ± 0.3	<0.0001
Ca^2+^ (mg/dl)	9.82 ± 0.1	8.83 ± 0.2	0.011
K^+^ (nmol/l)	4.37 ± 0.1	5.26 ± 0.2	<0.0001
Vitamin D (ng/ml)	47.63 ± 3.54	19.19 ± 1.19	<0.0001
Parathyroid hormone (pg/ml)	40.86 ± 2,01	505.9 ± 57.25	<0.0001
Alkaline phosphatase (U/l)	73.38 ± 5.62	81.38 ± 4.57	0.278

*Biochemical parameters are given as average ± SE in the indicated units. Statistical analysis was performed using Student's t-test*.

Serum biochemistry values are shown in Table 1 and are consistent with the expected differences between control and uremic serum for this stage of disease.

### Primary human aorta vascular smooth muscle cell culture and treatments

Primary human aorta vascular smooth muscle cells (HASMC) were isolated by ScienCell Research Laboratories, Inc. (Carlsbad, CA) and purchased through Innoprot (Derio, Spain). Primary HASMC were cultured according to the manufacturer's instructions. Briefly, cells were cultured at 37°C in a 5% CO_2_ atmosphere saturated with water. Culture medium was high glucose Dulbecco's Modified Eagles Medium (DMEM) supplemented with 10% fetal bovine serum, smooth muscle cell-specific growth factors (Innoprot), penicillin/streptomycin and 0.2% MycoZap™ (Lonza) to prevent mycoplasma contamination. Culture surfaces were pre-coated with a solution containing 1 mg/ml fibronectin in 0.2% gelatin (Sigma-Aldrich). Medium was replaced every 48 h until cells reached ~50% confluence. Afterwards medium was replaced daily and cells were subcultured when they reached 80–90% confluence. Once cells were confluent they were transferred to experimental medium consisting on culture medium supplemented with 20% of control or uremic human serum or DMEM with CaCl_2_, NaH_2_PO_4_, and Na_2_HPO_4_ to reach a final concentration of 2.5 mM inorganic phosphate (Pi) and 2 mM calcium in the medium. All experiments were performed using cells between passage 3 and 5.

### Calcification

Deposition of calcium phosphate crystals was assessed by alizarin red staining. Briefly, cells were seeded in 96-well plates and after reaching confluence were transferred to experimental media containing either control or uremic serum. Cells were then fixed for 30 min with 10% formalin. Cells were then washed and stained with 1% alizarin red (Sigma-Aldrich), which chelates calcium forming a red precipitate. The monolayers were washed twice with dH_2_O while shaking to remove the excess of dye. The plate was then immersed in 60% isopropanol and later in 100% isopropanol for 1 min. To quantify the amount of precipitate formed cells were dissolved with 10% acetic acid and the alizarin red absorption was measured at 450 nm in a plate spectrophotometer. Protein content was measured for each lysate using the bicinchoninic acid method (BCA assay kit, Sigma-Aldrich) following the manufacturer instructions.

### Alkaline phosphatase activity

Alkaline phosphatase activity, a marker for osteogenic activity, was quantified using a colorimetric assay in cell lysates. Briefly, cells were treated as described above, washed with PBS and lysed in a buffer containing 50 mM Tris pH6.8, 1% Triton X-100 and 2 mM MgCl_2_. After 30 min incubation, lysates were cleared by centrifugations and supernatants were used to determine enzyme activity. The reaction mixture (50 μl) included 50 mM p-nitrophenyl phosphate (pNPP, Sigma-Aldrich) prepared in 2-amino-2-methyl-1-propanol (Sigma-Aldrich). After 15 min of incubation at 37°C in darkness the reaction was stopped by addition of 3N NaOH. Conversion of pNPP to p-nitrophenol was quantified by reading the absorbance at 405 nm and normalization to total protein content measured by the BCA procedure (see above).

### Cell viability

HASMC viability was assessed using a colorimetric assay based on the reduction of the yellow tetrazolium salt XTT (2,3-bis(2-methoxy-4-nitro-5-sulfophenyl)-5-[(phenylamino)carbonyl]−2H- tetrazolium hydroxide) to an orange water-soluble formazan dye that only occurs in cells that are metabolically active (Scudiero et al., 1988). Viability was measured after 2, 5, or 10 days in experimental media using a commercial kit (Cell Proliferation Kit XTT, Applichem GmbH). Formazan dye concentration was measured by absorption spectrophotometry at 450 nm, repeating the measurement at 630 nm to subtract non-specific absorption. Viability is expressed as percentage of the value obtained under control conditions. HASMC cell viability was further studied by Trypan Blue exclusion and direct cell counting in a Neubauer chamber.

### Cell proliferation

HASMC proliferation was assessed by 5-ethynyl-2′-deoxyuridine (EdU) incorporation using a commercially available kit (Click-iT® EdU Imaging Kit, ThermoFisher Scientific) as we have described previously (Cidad et al., 2015). Briefly, cells were seeded at a density of 15,000 cells/cm^2^. One day after seeding cells were transferred to experimental media with control or uremic serum and incubated for 48 h. EdU was then added at a concentration of 10 μM and cells were incubated for 1, 6, 12, 24, or 48 h before fixation with 4% formaldehyde. Fluorescent staining of EdU with Alexa 488 fluorophore followed the manufacturer instructions. Cell nuclei were counterstained using Hoechst 33342 (ThermoFisher Scientific). Preparations were mounted using Mowiol (Calbiochem) containing 0.1% Diazobicyclo-octane (Aldrich) as anti-fading agent. Cells were examined under a Leica TCS SP8 confocal microscope. For quantitation, 15 independent fields were randomly selected and the proportion of cells with EdU positive nuclei was scored.

### Apoptosis

Quantification of apoptosis was performed by measuring cell surface accumulation of phosphatidylserine (PS) via binding of annexin-V-FITC (Santa Cruz Biotechnology) using flow cytometry. Briefly, treated cells were washed with PBS, centrifuged and resuspended in annexin-V binding buffer (in mM: 10 HEPES, pH 7.4; 140 NaCl; 2.5 CaCl_2_) supplemented with 0.2 μg/μl annexin-V-FITC. After 15 min of incubation cell were centrifuged and resuspended in the same buffer containing 0.6 μg/ml of propidium iodide (PI). Samples were analyzed in duplicate (15,000 cells/condition) in a MACSQuant Analyzer 10 apparatus (Miltenyi Biotec) using 488 nm light for excitation. FITC signal was detected at 518 nm and PI at 620 nm. Data are presented as percentage of cells undergoing early apoptosis (annexin-V-positive, PI-negative cells) or late apoptosis (annexin-V and PI-positive cells).

### Cell migration assays

To quantify the ability of HASMC to migrate in culture we used cultures grown to confluence in 35 mm dishes containing a silicon insert that forms two chambers with ~4 × 10^4^ cells each (Ibidi, GmbH). After 48 h in the presence of control or uremic serum, the silicon insert is removed and culture medium is changed to serum-free DMEM to prevent cell proliferation. Cells were then incubated for 24, 48, 72, or 120 h. Images were analyzed using ImageJ software (National Institutes of Health, Bethesda, MD) freely available at http://imagej.nih.gov/ij/ (Schneider et al., 2012). Migration is expressed as percentage of the initial empty area that remains free of cells.

### Quantification of gene expression

Total HASMC RNA was purified using a commercially available kit (Total RNA Spin Plus Kit, REAL, Valencia, Spain). Concentration and purity of RNA was estimated by using absorption spectrophotometry in a Nanodrop 2000 apparatus (Thermo Scientific). cDNA was synthesized using a commercial kit (iScriptTM cDNA Synthesis Kit, Bio-Rad) following the manufacturer instructions. Quantification of gene expression was performed using commercial Taqman® probes and a TaqMan® 2X Universal PCR Master Mix (Applied Biosystems, CA) in a CFX96 Touch™ Real-Time PCR Detection System (Bio-Rad). Each TaqMan probe was tested for linearity and efficiency of amplification, which was always between 90 and 110%. Normalization and relative quantification was performed with the ΔΔCt method (Schmittgen and Livak, 2008), using glyceraldehyde-3-phosphate dehydrogenase (GAPDH) as housekeeping gene.

### Immunocytochemistry

Indirect immunofluorescence staining was performed as previously described (Coric et al., 2004). Expression of smooth muscle actin (αSMA) was detected using mouse monoclonal antibody 1A4 (Sigma Aldrich) at 1:400 dilution. After staining cell were mounted in Mowiol medium containing 1 μg/ml DAPI to counterstain cell nuclei medium and examined under a Leica TCS SP8 confocal microscope.

### Intracellular calcium concentration measurements

Free intracellular Ca^2+^ concentration was measured using the fluorescent probe fluo-4. Cells were loaded with the probe using its acetoxymethyl esther (fluo-4/AM, Molecular Probes). Briefly, HASMC were seeded on glass coverslips and treated as described above. After washing in serum-free medium, cells were loaded with fluo-4/AM for 30 min at room temperature in the dark. Cells were then washed with complete medium to eliminate unincorporated probe and incubated for 10 more minutes at room temperature to allow for ester hydrolysis. Coverslips were then placed in a perfusion chamber in an inverted fluorescence microscope (Olympus IX70). A field of interest with several cells was selected and perfusion was started with extracellular solution (in mM: 10 HEPES, 141 NaCl, 4.7 KCl, 1.2 MgCl_2_, 1.8 CaCl_2_, 10 glucose, pH 7.4) and kept for 15 min in the dark to allow cells to stabilize. Experiments were performed using excitation light at 480 nm and recording through a dichroic mirror at 505 and an emission filter at 520 nm. Cells were treated with 10 μM angiotensin II to induce receptor-mediated intracellular Ca^2+^ transients. Cells were then perfused with Ca^2+^-free extracellular solution and treated with cyclopiazonic acid (10 μM) to inhibit sarco/endoplasmic reticulum Ca^2+^-ATPase (SERCA), emptying intracellular Ca^2+^ stores. At the end of experiment we added 10 μM ionomycin to make cells permeant to Ca^2+^ and obtain the maximum fluorescent signal (F_max_), which was used to normalize each experiment. Free Ca^2+^ concentration was calculated using the following equation:

$$\begin{array}{l} \left[Ca^{2+}\right]\;=300\;\times \;\left[\frac{\left(\frac{F480}{F480_{max}}\right)}{\left(\frac{1-F480}{F480_{max}}\right)}\right] \end{array}$$

where 300 (nM) is the Kd of Fluo-4 and F480 and F480_max_ are the fluorescence intensities of the stimulus and ionomycin respectively. Data recording and analysis was performed using Imaging Workbench 4.0 (Axon Instruments) and MatLab software, respectively.

### Statistical analysis

Statistical analysis was performed using Prism5 (GraphPad). Data are presented as average ± SE. Unless stated otherwise, each experiment was repeated at least three times and was performed in triplicate. Comparison between three or more groups was performed using one-way ANOVA followed by Tukey's *post-hoc* test. Comparison between two groups was performed using Student's *t*-test. Statistical significance was set at *p* < 0.05.

## Results

### HASMC develop calcification in response to uremic serum or increased extracellular phosphate

**Figure 1:**
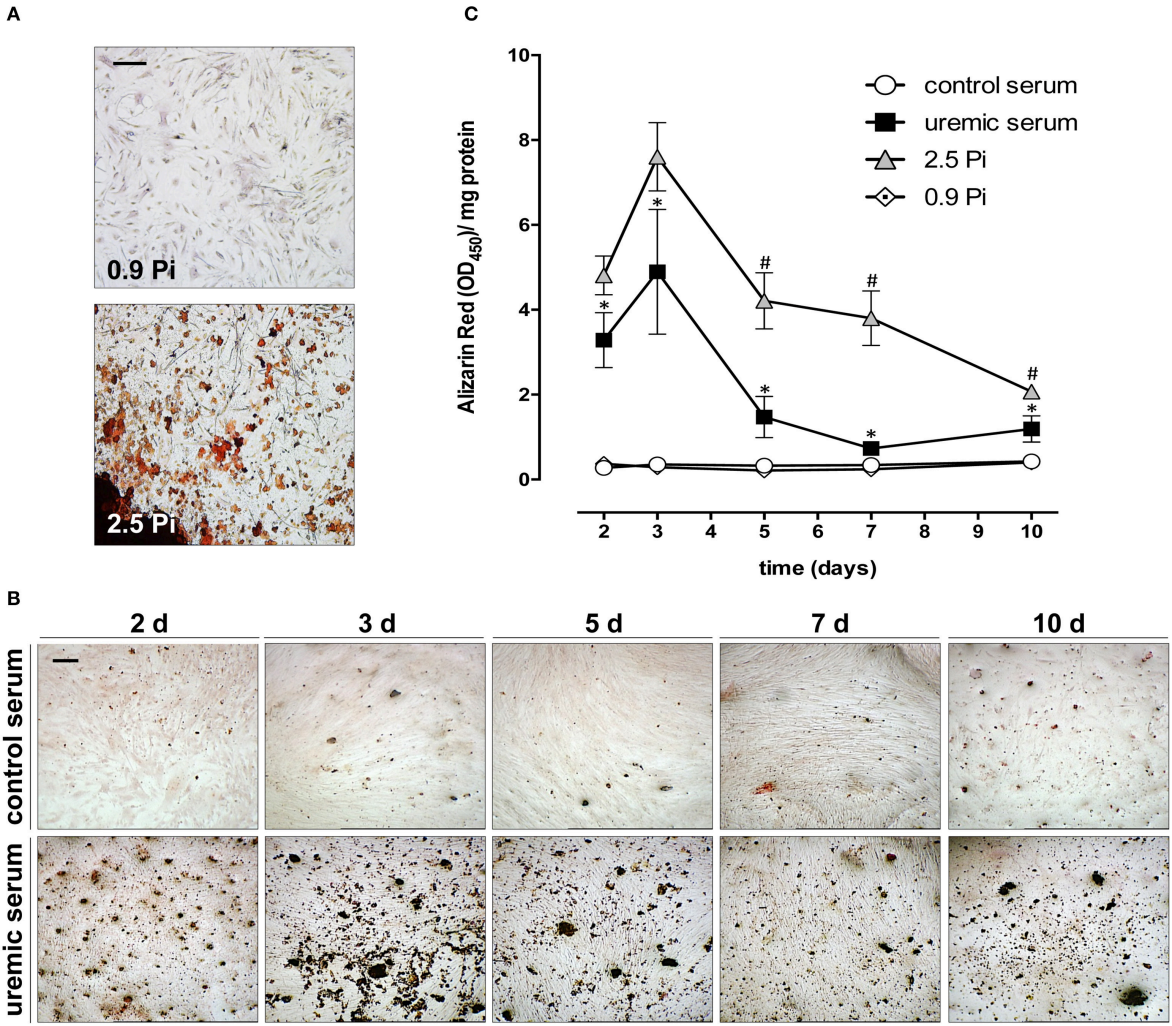
Quantification and time-course of calcification in HASMCs treated with high phosphate or serum from uremic patients. Cells were incubated for the indicated time in DMEM with the standard amount of inorganic phosphate (0.9 Pi), increased amount of Pi and Ca^2+^ (2.5 Pi), or in DMEM supplemented with 20% control or uremic serum. **(A)** Representative micrographs of HASMCs cultured in DMEM with 0.9 Pi or increased amount of inorganic phosphate and calcium (2.5 Pi) and stained with Alizarin red. Bar, 150 μm. **(B)** Representative micrographs of HASMCs cultured in DMEM supplemented with control or uremic serum for the indicated amount of times (values in days) and stained with Alizarin red. Bar, 110 μm**. (C)** Quantitative analysis of calcium phosphate deposition by Alizarin Red staining; each point represents the average ± SE absorbance at 450 nm for the indicated day of treatment (three independent experiments, with 4 replicas per condition in each experiment). Values were compared using a one-way ANOVA followed by Tukey's multiple comparisons test. ^*^Significant vs. control serum; ^#^significant vs. uremic serum.

We first tested the ability of uremic serum to induce calcification in primary cultures of human aortic vascular smooth muscle cells (HASMC). As a positive control, we also treated the cells with DMEM medium supplemented to bring Pi and Ca^2+^ concentration to 2.5 and 2 mM, respectively, a condition classically used to elicit calcification *in vitro*. Alizarin red staining revealed that increased extracellular Pi and Ca^2+^ induced extensive deposition of Ca^2+^-containing crystals, which are absent under normal conditions (Figure 1A). Colorimetric quantification of Ca^2+^ content revealed up to seven-fold accumulation after 3 days of treatment (Figure 1C). When HASMC where cultured with DMEM medium supplemented with human uremic serum they developed a similar pattern of calcium deposition, which peaked after 3 days of treatment (Figures 1B,C).

### Molecular markers of trans-differentiation are differentially regulated by Pi and uremic serum

**Figure 2:**
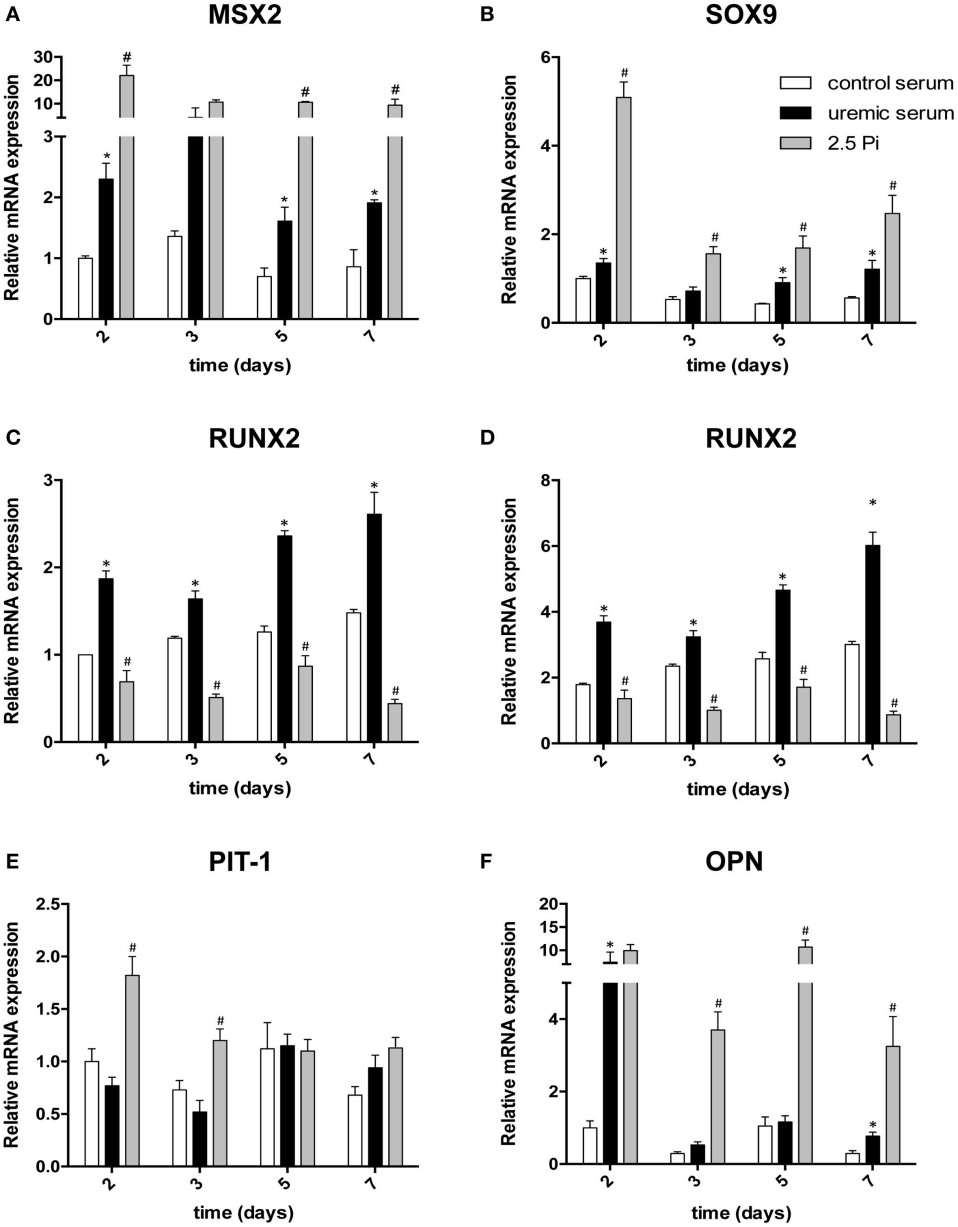
Differential modulation of osteochondrogenic differentiation marker expression in HAMSC treated with high phosphate or uremic serum. Cells were incubated for the indicated times in DMEM with increased Pi and Ca^2+^ (2.5 Pi) or in DMEM supplemented with 20% of control or uremic serum. Expression of the indicated osteochondrogenic markers (**A–F**; each individual marker is identified above the corresponding graph) was evaluated with qPCR. The expression of target genes was normalized to GAPDH. Results are presented as the mean ± SE (*N* = 3) normalized to expression under control serum conditions on day 2, except for **(D)**, where data are normalized to day 0. Values were compared using a one-way ANOVA followed by Tukey's multiple comparisons test. ^*^Significant change vs. control serum; ^#^significant change vs. uremic serum.

The previous experiment demonstrates that hyperphosphatemia and exposure to uremic serum produce similar calcification in HASMC. However, calcification associated to CKD involves HASMC trans-differentiation to an osteochondrogenic lineage. To investigate whether both calcification models produce similar trans-differentiation we examined the expression of molecular markers associated to this process. We first analyzed the expression of homeobox protein MSX2, a component of the bone morphogenetic protein pathway that promotes cardiovascular calcification (Tyson et al., 2003; Shimizu et al., 2012). Both Pi and uremic serum exposure induced the expression of MSX2 throughout the treatment (Figure 2A), with a stronger effect induced by the former. VSMC conversion to chondrocytes has also been implicated in medial calcification in uremic rats (Neven et al., 2010). Therefore, we quantified the expression of SOX9, a master regulator of chondrocyte differentiation (Liu C.F. et al., 2016). SOX9 expression followed a pattern similar to MSX2, with both stimuli enhancing expression throughout the treatment, with a more marked induction produced by Pi (Figure 2B). Runt-related transcription factor 2 (RUNX2), a transcription factor that is essential for osteochondrogenic differentiation, was significantly enhanced throughout the treatment with uremic serum (Figure 2C). Surprisingly, hyperphosphatemia repressed the expression of RUNX2 (Figure 2C), suggesting that this model produces a pattern of HASMC trans-differentiation that is significantly different from that induced by uremia. Given that increased RUNX2 expression is a transient early event in osteoblast differentiation, we also compared its level to expression at day 0 of the experiment (Figure 2D). It can be observed that control human serum increases RUNX2 expression, but to a lower extent than uremic serum.

The result obtained with RUNX2 expression prompted us to investigate further markers involved in osteochondrogenic differentiation. One such marker, the Na^+^/Pi co-transporter Pit-1 has been proposed to play an important functional role in this process in a rat model of uremia (Mizobuchi et al., 2006) and in phosphate-induced calcification of cultured human VSMC (Li et al., 2006). Consistently with this report, we found induction of Pit-1 mRNA in HASMC exposed to hyperphosphatemia at every time point examined, starting after 2 days of treatment (Figure 2E). In contrast, treatment with uremic serum only induced increased Pit-1 expression after 7 days, which does not correlate with the start of calcification. This result also suggests that the trans-differentiation mechanisms induced by hyperphosphatemia and uremia may be fundamentally different.

An important component modulating tissue biomineralization is the expression of calcification inhibitors such as osteopontin (OPN, also known as secreted phosphoprotein 1, or SPP1), which acts by regulating crystal growth (Wada et al., 1999; Lomashvili et al., 2004). Upregulated expression of OPN has been demonstrated at sites of ectopic calcification, including the vascular wall (Abedin et al., 2004). We tested OPN mRNA expression in HASMC and found a potent initial up-regulation both by uremic serum or high extracellular phosphate. However, uremic serum effects OPN were mostly transient, while increased extracellular phosphate maintained a high level of OPN expression throughout the experiment (Figure 2F).

**Figure 3:**
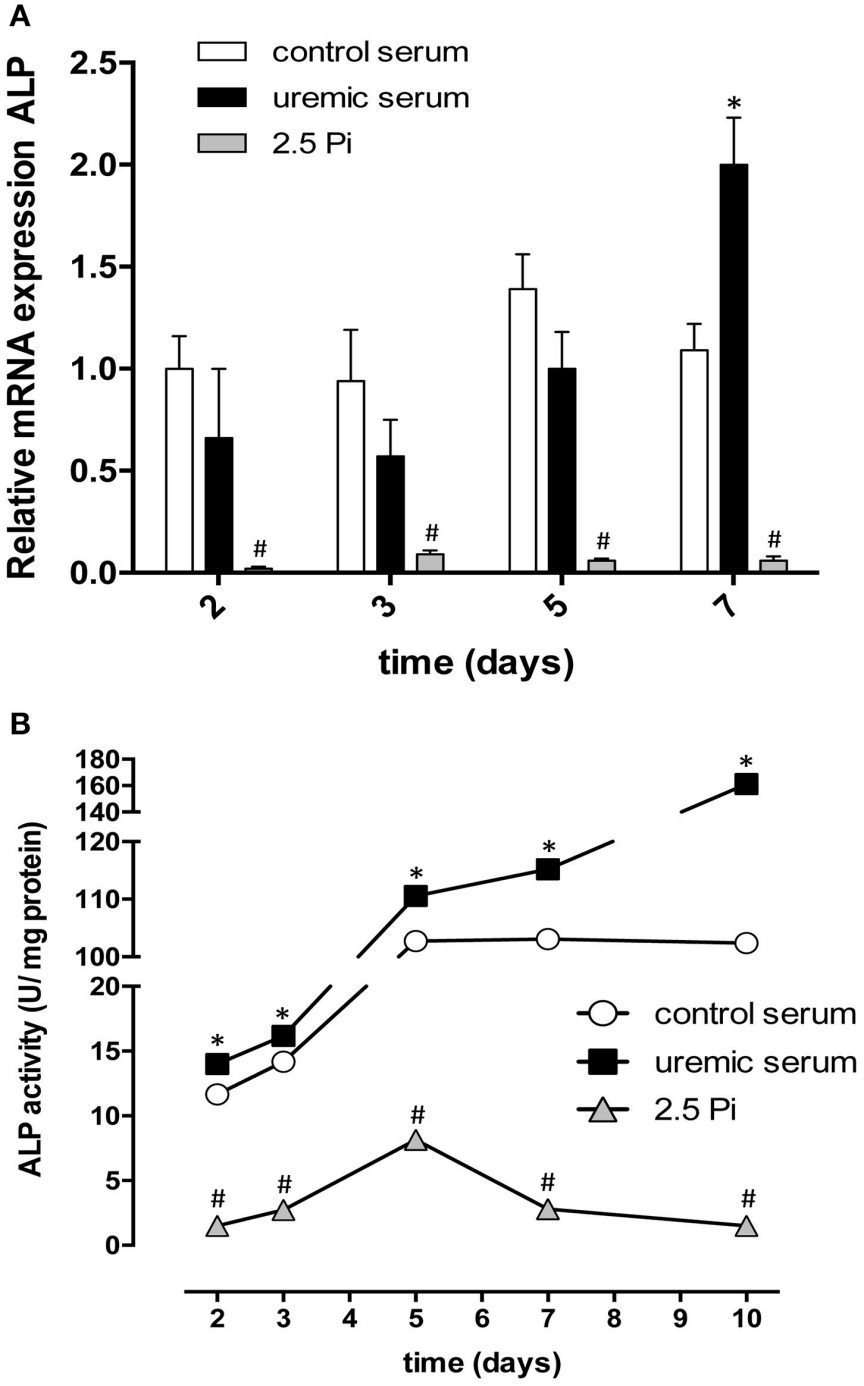
Expression and activity of alkaline phosphatase are increased by uremic serum but potently reduced by high phosphate. **(A)** Alkaline phosphatase (ALP) mRNA expression in HASMC incubated in DMEM for the indicated amount of time with increased Pi and Ca^2+^ (2.5 Pi), or in DMEM supplemented with 20% control or uremic serum. The expression of ALP gene was normalized to GAPDH. Results are presented as the mean ± SE (*N* = 3); ^*^significant vs. control serum; ^#^significant vs. uremic serum. **(B)** Intracellular ALP activity measured using a colorimetric assay. Each measurement was normalized for total protein. Data are shown as mean ± SE (*N* = 3). Values were compared using a one-way ANOVA followed by Tukey's multiple comparisons test. ^*^Significant vs. control serum; ^#^significant vs. uremic serum.

One of the most relevant markers of osteoblast activity is alkaline phosphatase (ALP). We studied ALP expression and function in control HASMC or cells treated with uremic serum or exposed to increased Pi. Surprisingly, exposure to high extracellular Pi potently repressed ALP expression and activity (Figures 3A,B). In contrast, uremic serum did not alter ALP expression during the first 5 days of the experiment, but produced a significant increase in mRNA abundance in cells exposed to uremia for 7 days (Figure 3A). Most importantly, ALP activity was potently repressed by Pi but significantly increased by uremic serum for the duration of the experiment (Figure 3B).

### High phosphate dramatically decreases HASMC viability

**Figure 4:**
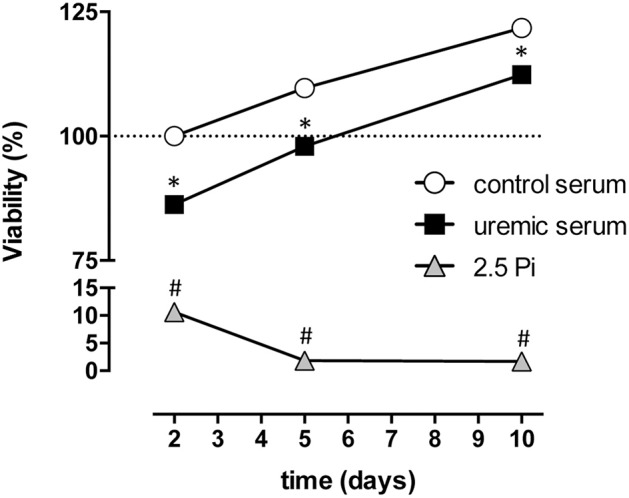
Contrasting effects of high phosphate and uremic serum on HASMC viability. Cells were cultured either in DMEM supplemented with high phosphate (2,5 Pi) or 20% of control or uremic serum. Cell viability was assayed after 2, 5, and 10 days using the 2,3-Bis-(2-methoxy-4-nitro-5-sulfophenyl)-2H-tetrazolium-5-carboxanilide salt (XTT) viability test. Values are expressed as the mean ± SE (*N* = 3) and compared using a one-way ANOVA followed by Tukey's multiple comparisons test; ^*^significant vs. control serum; ^#^significant vs. uremic serum.

**Figure 5:**
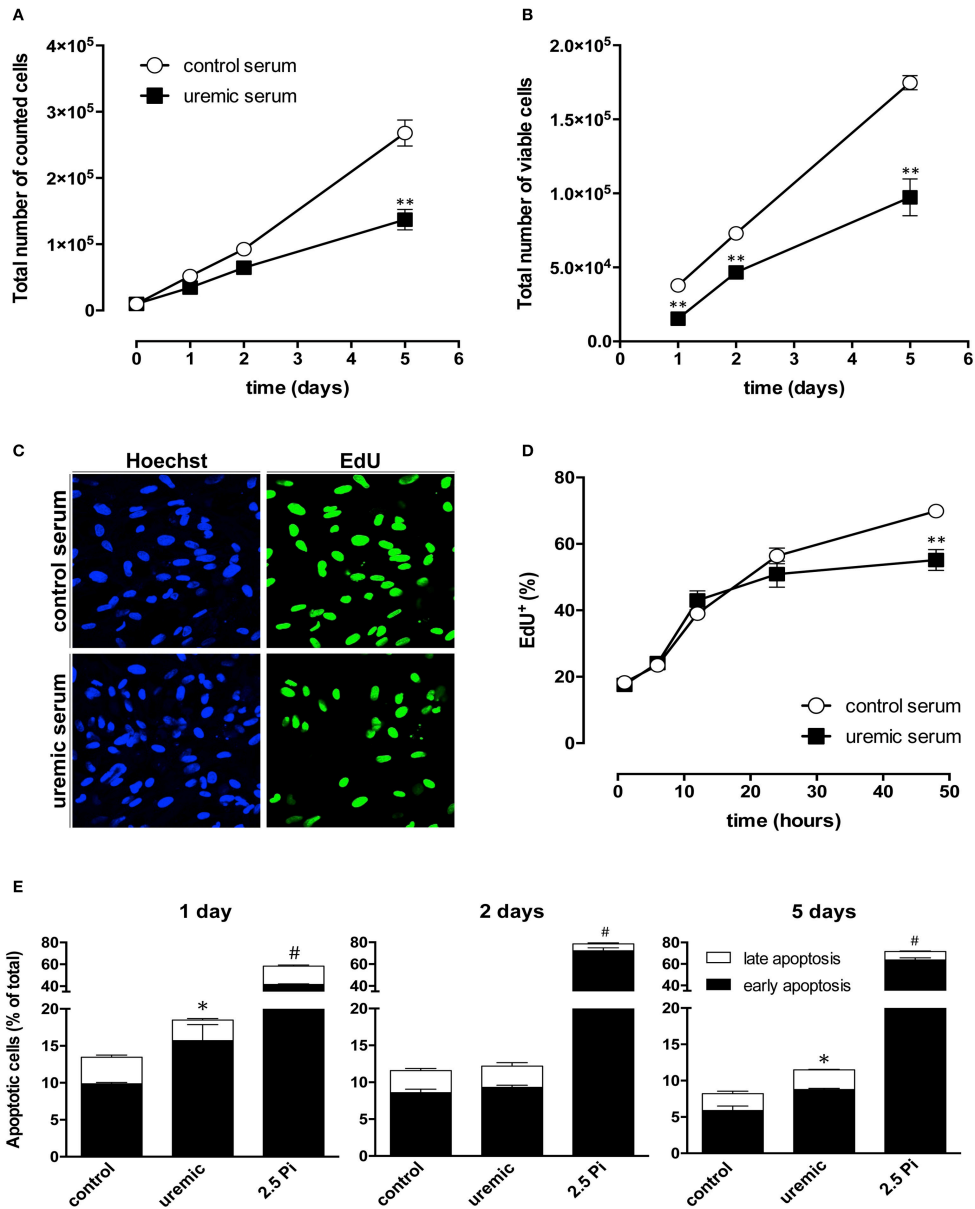
Uremic serum-exposed HASMC show slightly decreased cell proliferation and increased apoptosis. **(A)** Total number of cells in cultures exposed to control or uremic serum for the indicated amount of time. Bars represent mean ± SE (*N* = 3). (**B**) Number of Trypan blue-excluding (viable) cells in cultures exposed to control or uremic serum for the indicated amount of time. Bars represent mean ± SE (*N* = 3). **(C)** HASMC cell proliferation was measured using the EdU incorporation test. Proliferating cells have green nuclei and were counterstained with Hoechst 33342. **(D)** Quantification shows mean ± SE of proliferating cells (*N* = 3). Student's *t*-test, ^**^P < 0.01. **(E)** Annexin V apoptosis assay using flow cytometry analysis in HASMC cells cultured in DMEM supplemented with high phosphate (2,5 Pi) or 20% of control or uremic serum. Data represent the percentage of apoptotic cells analyzed with annexin V-FITC/PI flow cytometry at the indicated days of treatment. Data represent the percentage of cells in early (annexin V positive, PI negative cells; black portion of each bar) or late apoptosis (positive for both markers; white portion of each bar). Values are mean ± SE (*N* = 3) and compared using one-way ANOVA followed by Tukey's multiple comparisons test; ^*^significant vs. control serum; ^#^significant vs. uremic serum.

In order to compare the effect of high Pi with uremic serum on cell viability we tested HASMC ability to reduce a tetrazolium salt (XTT) in the presence of an electron-coupling reagent, to produce a water-soluble formazan dye. This reaction depends on NAD(P)H, which in turn depends on cellular metabolic activity. XTT reduction was measured at 2, 5, and 10 days of treatment. In both control and uremic conditions the amount of XTT, which is proportional to cell proliferation and viability, increased over time (Figure 4). Uremic serum induced a small but significant decrease in XTT reduction, which averaged 9–13% less than in control cells, a difference that tended to decrease over time (Figure 4). In contrast, high Pi produced a dramatic decrease in cell viability, which reached a 90% decline during the course of the experiment (Figure 4). Together with the data examining the expression of trans-differentiation markers, these results indicate that exposing HASMC to high phosphate medium not only does not reproduce the expected differentiation process, but also decreases massively cell viability. This strongly indicates that using high phosphate as a model for uremic calcification is inadequate. Since XTT reduction depends both on cellular metabolic activity, which is related to cell viability, and also on the number of cells present on the culture, we directly examined whether uremic conditions alter cell proliferation and decrease the overall number of living cells in culture. To that end we measured the total number of viable HASMC in culture by counting cells able to exclude Trypan Blue. Total number of cells in culture did not change until after 5 days of treatment with uremic serum (Figure 5A). When only viable cells are taken into account, uremia induced a slight decrease in every time point tested (Figure 5B). Experiments using Pi treatment were difficult to interpret, most likely due to the dramatic decrease in cell viability detected under this condition. It is worth noting that the decrease in viable cells induced by uremic serum and assessed by Trypan Blue exclusion (~50%) is much higher than that estimated by XTT reduction (Figure 4). This result suggests that in the presence of uremic serum cells proliferate at a lower rate but may have a higher than normal metabolic activity, as assessed by XTT reduction, which in turn depends on NAD(P)H availability.

To directly assess HASMC proliferation rate we measured EdU incorporation as readout of DNA synthesis (Figure 5C). EdU incorporation progressively increased with time, as expected (Figure 5D). Uremic serum did not have a significant effect during the first day of treatment, but decreased EdU incorporation by 15% after 2 days.

Taken together, the data suggests an early decrease in cell viability, followed by slightly decreased proliferation by day 2, which may be accompanied by higher metabolic activity in the surviving cells. To confirm this observation we directly assessed the occurrence of apoptosis in control, Pi-treated or uremic serum-treated HASMC cultures using flow cytometry in cells stained with annexin V and propidium iodide (PI). Annexin V-positive and PI-negative staining was considered a sign of early apoptosis, while positive staining for both markers was considered a sign of late apoptosis/necrosis. After 1 day-treatment with uremic serum, there was an overall increase in apoptotic cells in culture from ~13 to 18%. This increase was mostly due to enhanced early apoptosis, which changed from ~10 to 15% (Figure 5E). This is in agreement with the data obtained with our proliferation measurements, which suggest an early decrease in cell viability. After 2 days in uremic serum, HASMC did not show any significant difference in early or late apoptosis when compared to control cells (Figure 5E), again confirming the proliferation data. By day 5 of treatment apoptosis is generally low in both conditions, although slightly increased in uremia-exposed cells (Figure 5E). Consistently with the dramatic loss of cell viability detected with Pi, this treatment produced a very large increase in cell apoptosis starting as soon as day 1 (Figure 5E). Therefore, the rest of our study focused on treating cells with uremic serum.

### Uremic serum decreases smooth muscle actin expression and impairs calcium handling in HASMC

**Figure 6:**
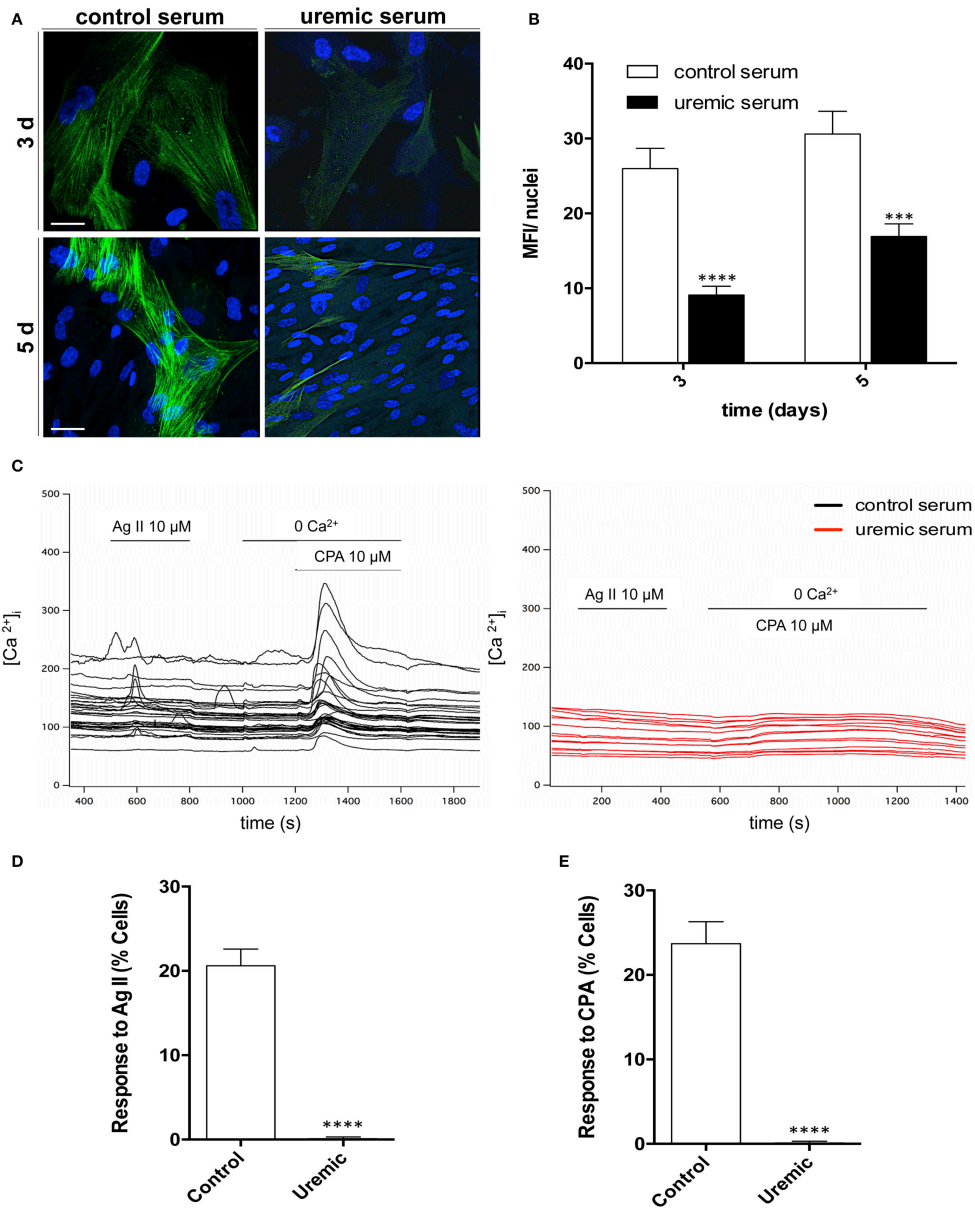
Uremia decreases expression of contractility markers, blunts receptor-operated channel response and depletes intracellular Ca^2+^ stores. **(A)** Representative micrographs of HASMC treated with control or uremic serum, fixed, and stained with anti-α-SMA antibody (green). Nuclei were counterstained with DAPI (blue). Bar, 28 μm. **(B)** Quantitative analysis of α-SMA expression. Bars present average fluorescent intensity normalized by nuclei number ± SE obtained from 15 different fields in two independent experiments. **(C)** Representative traces of Fluo4-loaded HASMC pre-treated with control or uremic serum, stimulated with 10 μM angiotensin II (AngII) and then switched to Ca^2+^-free extracellular solution and stimulated with 10 μM of cyclopiazonic acid (CPA). Traces represent cytosolic Ca^2+^ concentration (in nM) over a period of 1,400 s. **(D)** Quantitative analysis of cells responding to AngII stimulation with a rise in cytosolic Ca^2+^. Bars represent average ± SE. **(E)** Quantitative analysis of cells responding to CPA treatment. Bars represent average ± SE. Student's *t*-test: ^***^P < 0.001, ^****^P < 0.0001.

In order to examine the consequences of uremic serum on the functional properties of HASMC, we first measured expression of smooth muscle α-actin, the actin isoform characteristically expressed in VSMC (Skalli et al., 1986). Immunocytochemistry revealed robust expression in control cells, with the characteristic fibrilar pattern (Figure 6A). Uremic serum produced a marked decrease in smooth muscle α-actin expression that was already apparent after 3 days of treatment (Figures 6A,B).

Given that the response of VSMCs to physiological stimuli depends on a rise in cytosolic Ca^2+^, we next examined the effect of uremia on Ca^2+^ handling. Average basal cytosolic Ca^2+^ concentration decreased after treatment with uremic serum (in nM: 133.0 ± 6.3 in control cells, 103.7 ± 5.2 in uremia-exposed cells; Figure 6C). Response to a known vasoconstrictor agent such as angiotensin II was blunted in uremic cells (Figures 6C,D). Treatment with cyclopiazonic acid, a known inhibitor of SERCA, increased cytosolic Ca^2+^ in ~25% of control cells, but failed to elicit a response in cells treated with uremic serum, suggesting total depletion of intracellular Ca^2+^ stores (Figures 6C,E). Of note, in spite of their lack of response, uremia- treated cells are able to maintain a low cytosolic Ca^2+^ concentration, implying that the mechanisms that actively extrude Ca^2+^ are functional in those cells. Altogether, the data indicate that, in spite of the moderate changes observed in cell viability and proliferation, uremic serum treatment leads to a dramatic impairment of the functional responses of HASMC.

### Uremic serum decreases HASMC migration

**Figure 7:**
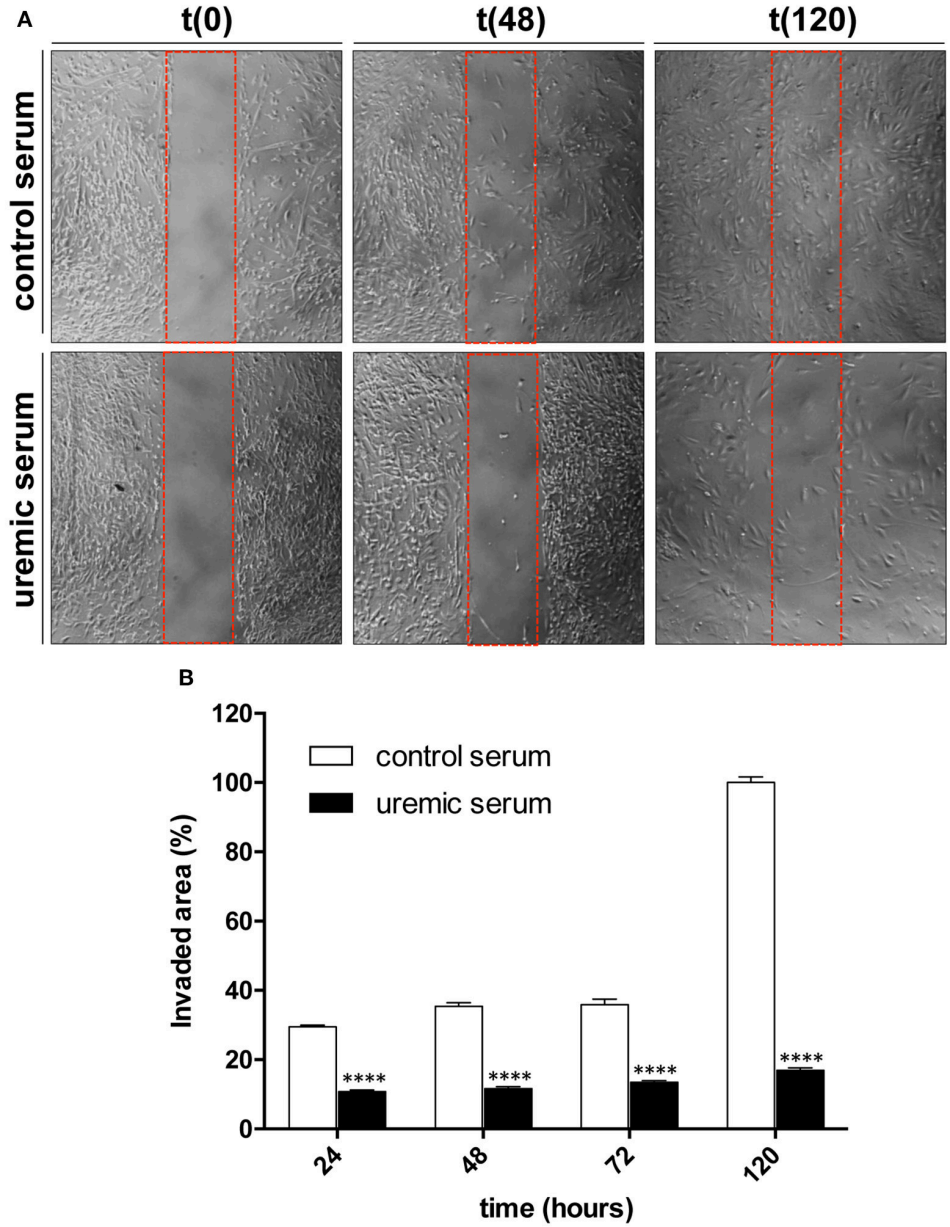
Uremia decreases migration of cultured HASMC. **(A)** Cells were grown to confluence in 35 mm dishes containing a silicon insert forming two chambers while treated with DMEM supplemented with control or uremic serum. After removal of the insert culture medium was changed to serum-free DMEM to prevent cell proliferation. Cells were then monitored and photographed at the indicated times with a phase-contrast microscope. **(B)** Values represent percentage of invaded area (mean ± SE, *N* = 3). Student's *t*-test: ^****^P < 0.0001.

Disease-associated phenotypic changes in VSMC not only produce an change in proliferation but also affect the capacity of the cells to migrate, a feature that has been propose to participate in the development of intimal hyperplasia (Schwartz, 1997). Therefore, we used cultured HASMC to assess whether uremia alters cell migration. To that end we used cells grown on culture dishes containing a silicon insert that creates a well-defined void. Cells were pre-treated with control or uremic serum for 48 h and then the silicon insert was removed and culture medium changed to serum-free conditions to prevent cell proliferation. Control cells progressively filled the void, reaching confluence after 5 days (Figure 7A). In contrast, HASMC pre-treated with uremic serum displayed low migration capacity, reaching less that 20% confluence on the void space in the same time frame (Figure 7B). Surprisingly, almost all of the migration in uremic conditions took place in the first day of incubation, which suggests that cells exposed to uremic serum retain a certain degree of migration ability, but the treatment pre-conditions them to maintain migration rates at a very low level (Figures 7A,B).

## Discussion

A very common model of *in vitro* calcification of VSMC cultures is the use of high extracellular Pi, which simulates the hyperphosphatemia detected in late stages of CKD (Jono et al., 2000). However, this model fails to take into account the complex metabolic and hormonal alterations present in uremic patients. In addition, a large number of published studies used VSMC obtained from bovine or rat aorta, which may produce conflicting results due to species-specific differences in the process. Therefore, we sought to examine the process of mineralization and phenotypic remodeling in primary human aortic VSMC comparing changes elicited by high extracellular Pi with those produced by uremic serum obtained from CKD patients. Our results show that exposure to uremic serum induces mineralization of HASMC in culture, along with increased expression of osteogenic markers and increased alkaline phosphatase activity. The extent of mineralization is lower than that induced by Pi, but the kinetics was similar. Surprisingly, the peak accumulation of calcium was detected at day 3, with partial reversal afterwards. We did not investigate the mechanism behind this phenomenon, but it suggests that the use of this model is not adequate for studying chronic aspects of vascular remodeling associated with uremia, but rather short-term effects of uremic serum. This idea is reinforced by the fact that isolated cultured HASMC do not reproduce the microenvironment of the vessels, where the cells are influenced by endothelial factors, the interaction with the extracellular matrix and by hemodynamic variables.

The ability of uremic serum to induce calcification in cultured SMC was already suggested by Chen et al. who demonstrated that uremic serum supplemented with β-glycerophosphate, insulin and ascorbic acid induced calcification in bovine VSMC (Chen et al., 2006b). Our results are also in agreement with the findings of Liu Y. et al. (2016) using a similar model (HASMC treated with 10% control or uremic serum pooled from stage 5 patients). However, these studies did not compare the effect of patient serum with hyperphosphatemia. Remarkably, our results show that expression of osteochondrogenic markers and other genes involved in the development of biomineralization is fundamentally different between both stimuli. In addition, Pi induces calcification without measurable increases in alkaline phosphatase expression or activity, casting doubts on the physiological relevance of this model. Most likely, massive loss of viability and increased apoptosis in cells treated with Pi is probably mediating passive Pi precipitation. In contrast, the effect of uremic serum in cell viability and apoptosis is only moderate, although statistically significant respect to control serum. This reduction of cell viability directly correlates with a decrease in cell number, and might be explained by decreased proliferation, increased apoptosis or a combination of both mechanisms. A close look at the kinetics of uremic serum effects on the number of cells in culture reveals a decrease as soon as 24 h after starting the treatment, an effect that is apparent also with Pi, correlating well with increased apoptosis within the same time frame. In comparison, Hortells et al. measured lactate dehydrogenase release as a measure of cell death induced by Pi in rat VSMC and found that 3 mM Pi increased cell death only after 4 days of treatment (Hortells et al., 2015). This difference may reflect the different species source used (rat vs. human in our study) or other differences in cell culture procedure. Regarding cell proliferation, a decrease is only apparent after 48 h of treatment. Taken together, these results suggest that the effect of uremic serum on the number of cells in culture is due to a combination of increased apoptosis and decreased proliferation, with varying relative importance at different times of treatment. It has been previously described that calcification is linked to increased apoptosis of VSMCs (Proudfoot et al., 2000; Shroff et al., 2008), but not in mesenchymal stem cells, where uremic serum-induced calcification was not accompanied by increased apoptosis (Kramann et al., 2011).

In addition to proliferation, it has been proposed that altered VSMC migration contributes to intimal lesions in patients with CKD (Schwartz, 1997). Therefore, we studied VSMC migration in our model and found that, contrary to the situation *in vivo*, uremic serum produced a marked decrease in the capacity of VSMC to migrate. A previous study also examined the effects of uremic serum on VSMC migration and found that uremic serum inhibited PDGF-induced VSMC migration (Monroy et al., 2015). These results suggest that *in vitro* models of cultured VSMC are not appropriate for studying uremia-induced alterations in cell migration. This may arise from the fact that uremic serum used in these models is diluted in culture medium, but could also be due to the lack of additional signaling molecules derived from the endothelium or induced by the three-dimensional structure of the vascular wall.

In summary, our data reveals important differences in the molecular events leading to phenotypic remodeling and trans-differentiation of human VSMC exposed to Pi or uremic serum and suggests that the latter provides a more informative model for the study of vascular calcification and at least some aspects of phenotypic remodeling secondary to CKD. This model, in addition to others such as *in vitro* differentiation of mesenchymal stem cells to osteoblasts (Hoemann et al., 2009) should shed light on the complex process of vascular calcification associated to CKD.

## Author contributions

VC-P: Performed experiments, analyzed data, interpreted results, prepared figures, and participated in drafting the manuscript. PC and MP-G: Performed experiments, analyzed data and interpreted results; JD-C and EM-N: Performed determinations on serum samples and analyzed data; JL-L: Analyzed data and interpreted results of experiments; TG: Participated in the study design, analyzed data, interpreted results, and participated in drafting the manuscript; JN-G: Selected the patients, participated in the study design, interpreted results, and participated in drafting the manuscript; DA: Participated in the study design, analyzed data, interpreted results, and drafted the manuscript. All authors revised the manuscript and approved its final version.

### Conflict of interest statement

The authors declare that the research was conducted in the absence of any commercial or financial relationships that could be construed as a potential conflict of interest.
